# Serotyping and Evaluation of Antimicrobial Resistance of *Salmonella* Strains Detected in Wildlife and Natural Environments in Southern Italy

**DOI:** 10.3390/antibiotics10040353

**Published:** 2021-03-27

**Authors:** Immacolata La Tela, Maria Francesca Peruzy, Nicola D’Alessio, Fabio Di Nocera, Francesco Casalinuovo, Maria Rosaria Carullo, Davide Cardinale, Daniela Cristiano, Federico Capuano

**Affiliations:** 1 Salmonella Typing Centre of the Campania Region, Department of Food Microbiology, Istituto Zooprofilattico Sperimentale del Mezzogiorno, Portici, 80055 Naples, Italy; immacolata.latela@cert.izsmportici.it (I.L.T.); mariarosaria.carullo@cert.izsmportici.it (M.R.C.); 2 Department of Veterinary Medicine and Animal Production, University of Naples “Federico II”, 80137 Naples, Italy; mariafrancesca.peruzy@unina.it; 3 Department of Animal Health, Istituto Zooprofilattico Sperimentale del Mezzogiorno, Portici, 80055 Naples, Italy; nicola.dalessio@cert.izsmportici.it (N.D.); fabio.dinocera@izsmportici.it (F.D.N.); 4 Diagnostic Unit of Catanzaro, Istituto Zooprofilattico Sperimentale del Mezzogiorno, 88100 Catanzaro, Italy; francesco.casalinuovo@cert.izsmportici.it; 5 Diagnostic Unit of Avellino, Istituto Zooprofilattico Sperimentale del Mezzogiorno, 88024 Monteforte I., Italy; davide.cardinale@izsmportici.it; 6 Department of Food Microbiology, Istituto Zooprofilattico Sperimentale del Mezzogiorno, Portici, 80055 Naples, Italy; daniela.cristiano@izsmportici.it

**Keywords:** *Salmonella* serovars, wildlife, antimicrobial resistance

## Abstract

Wild animals are potential vectors of antibiotic-resistant bacteria in the environment. The present study aimed to investigate the occurrence of antimicrobial resistance among *Salmonella* serovars isolated from wildlife and the environment in Italy. A total of 164 *Salmonella* isolates were analyzed, and six different subspecies and 64 serovars were detected. High proportions of *Salmonella* isolates proved resistant to streptomycin (34.1%), followed by trimethoprim-sulfamethoxazole (23.2%), tetracycline (17.7%), ciprofloxacin (14.63%) and ampicillin (11.59%). By source, the lowest level of resistance was observed in *Salmonella* serovars isolated from a water environment, while antimicrobial resistance was frequent in strains collected from shellfish, reptiles and birds. Multidrug-resistant strains were recovered from seafood (*n* = 11), mammals (*n* = 3) and water (*n* = 1). Three *S*. Typhimurium monophasic variant strains showed asimultaneous resistance to ampicillin, streptomycin, tetracycline and trimethoprim-sulfamethoxazole, which represents a recognized alert resistance profile for this serovar. These data indicate the environmental dissemination of resistant strains due to anthropogenic activities, which, in southern Italy, probably have a higher impact on marine ecosystems than on terrestrial ones. Moreover, as most of the animals considered in the present study are usually consumed by humans, the presence of resistant bacteria in them is a matter of great concern.

## 1. Introduction

*Salmonella* are Gram-negative bacteria belonging to the Enterobacteriaceae family. The genus *Salmonella* includes two species, *Salmonella* (*S*.) *bongori* and *S. enterica*, and six subspecies: *S*. *enterica* subsp. *arizonae*, *S*. *enterica* subsp. *diarizonae*, *S*. *enterica* subsp. *enterica*, *S*. *enterica* subsp. *houtenae*, *S*. *enterica* subsp. *indica* and *S*. *enterica* subsp. *salamae* [1]. Moreover, the subspecies *enterica*, according to its surface antigens (O and H), can be divided into over 2600 serovars [2].

*Salmonella* is an enteric pathogen that colonizes the intestinal tract of a wide range of animals, including not only primates, livestock, birds and pets, but also cold-blooded animals and wild fauna [3]. Wildlife plays a complex and important role in the maintenance and transmission of this pathogen and those that cause other endemic diseases [4,5]. Although animals may develop diseases such as enteritis and septicemia, and suffer abortion, *Salmonella* infections in animals are generally asymptomatic [6]. Infected animals may excrete *Salmonella* bacteria in large numbers, spreading the pathogen to other habitats, such as water, foodstuffs and the environment, in which it can survive for a long period [7]. It has been reported that wild animals can act as reservoirs of different *Salmonella* serotypes [3], which may be transmitted both to domestic animals and to humans [8]. The transmission of *Salmonella* among humans, domestic animals and wildlife mainly occurs through direct contact with live animals or the consumption of contaminated food or water [9]. *Salmonella* infection in humans is usually associated with raw eggs and inadequately cooked meat. Although the consumption of wild animals (mammals, birds, reptiles and amphibians) is still far lower than that of domestic animals, it is increasing worldwide [10]. Thus, the presence of *Salmonella* in wild animals may constitute a great risk for public health.

Moreover, the impact of *Salmonella* infections has increased in recent decades because of the rapid emergence of antibiotic-resistant bacteria (ARB) worldwide. Over the years, the extensive use and misuse of antibiotics in human and veterinary medicine, agriculture and aquaculture has led to the spread of resistant bacteria, which already cause 700,000 deaths each year [11,12]. Salmonellosis is one of the most common zoonoses in humans in the European Union (EU) [13], and is the leading food-borne disease in Italy. In humans, infections are generally self-limiting and do not require antimicrobial treatment [14], but in rare cases the infection can be more serious, necessitating the use of antimicrobial agents. However, owing to the increased resistance of *Salmonella* spp., severe infections are often difficult to treat.

Wildlife may spread ARB in the environment via their feces [9]. The spread of resistant *Salmonella* strains in the natural environment constitutes a potential hazard for both humans and animals [15].

Italy is a densely populated country with considerable biodiversity and wildlife populations. Few data are available on the distribution of *Salmonella* serovars and the occurrence of antimicrobial resistance among them in wild animals, except for a few species (such as some birds and wild boars) [6,15]. Monitoring *Salmonella*-resistant strains in wild animals may constitute an important means of determining the level of dissemination of resistant strains in the environment. The present study therefore aimed to investigate the occurrence of antimicrobial resistance among *Salmonella* serovars isolated from wildlife.

## 2. Results

### 2.1. Serotyping

The isolates were assigned to the species *Salmonella enterica* and to the subspecies: *enterica* (82.3%), *diarizonae* (9.1%), *salamae* (5.5%), *houtenae* (1.2%), *arizonae* (1.2%) and *indica* (0.6%) (Table 1).

Shellfish, snails and amphibians harbored strains belonging exclusively to a single *Salmonella* subspecies, while the remaining sources harbored *Salmonella* isolates belonging to three to six subspecies (Table 1). The number of *S. enterica* strains isolated from cold-blooded animals (amphibians, snails and reptiles) was significantly lower than the number isolated from warm-blooded animals (birds and mammals) and shellfish (*p* < 0.05). Serotyping identified 64 serovars (Table 2); *S.* Napoli was the most frequently detected (13 isolates), followed by *S*. Typhimurium (11 isolates), *S*. Enteritidis, *S*. Rissen (9 isolates each), and *S*. Derby (8 isolates).

### 2.2. Antibiotic Susceptibility

Overall, 60 isolates (36.6%) showed susceptibility to all antibiotics tested. The number of isolates susceptible to all antibiotics was significantly lower among the strains belonging to non-*enterica* subspecies than among those belonging to the *enterica* subspecies (*p* < 0.05), but with an average number of resistances of 2.13 and 1.6 for *S*. *enterica* and *S*. non-*enterica*, respectively. High levels of resistance were observed against aminoglycosides (35.9%), followed by quinolones (20.7%) and beta-lactams (15.8%) (Table 3).

**Figure 1:**
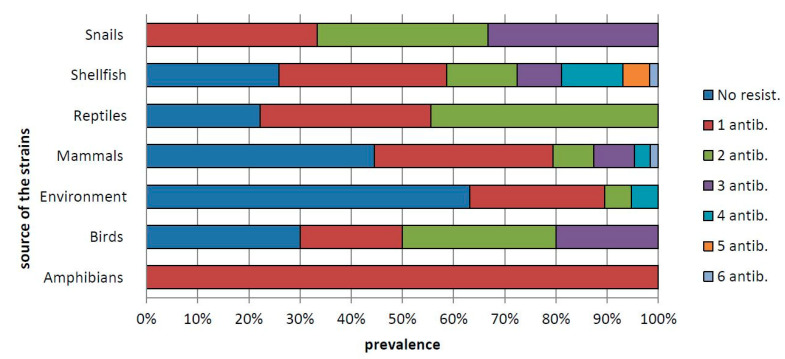
Percentage of *Salmonella* strains resistant to one or more (up to six) antibiotics in isolates from amphibians (frogs), birds, environment, mammals, shellfish, reptiles and snails.

Among the single antimicrobial agents tested, the highest frequency of resistance was toward streptomycin (34.1%), followed by trimethoprim-sulfamethoxazole (23.2%), tetracycline (17.7%), ciprofloxacin (14.63%) and ampicillin (11.59%), while the lowest levels of resistance were against ceftazidime (1.8%) and colistin sulfate (1.2%) (Table 3). In total, 54 of the resistant strains (32.79%) were resistant to only one antibiotic; 22 strains (13.4%) showed resistance to two antibiotics, and the remaining strains showed resistance to three to six antibiotics (Figure 1).

By source, the lowest level of resistance was observed in *Salmonella* serovars isolated from water (environment), while antimicrobial resistance was frequent in strains collected from shellfish, reptiles and birds (Table 3). On comparing the occurrence of resistant and susceptible strains in the various sources, the number of strains resistant to one or more antimicrobial agents was significantly higher in shellfish (*p* < 0.05). In particular, the strains detected in shellfish showed significantly higher resistance toward ampicillin and tetracycline (*p* < 0.05). Overall, 43 resistance profiles (R-types) were detected (Table 4).

Four profiles of antibiotic resistance were common to several subspecies of *Salmonella*. In total, 37 of these R-types were found in isolates of *Salmonella* subspecies *enterica* (27.41%), 7 R-types in *S*. *salamae* (77.78%), 5 in *S*. *diarizonae* (33.33%), 2 in *S*. *houtenae* (100%), and 1 R-type each in *S*. *arizonae* (50%) and *S*. *indica* (100%). Among the enterica subspecies, 13 strains showed simultaneous resistance to ampicillin and trimethoprim-sulfamethoxazole, 11 to ampicillin, tetracycline and trimethoprim-sulfamethoxazole, 7 to ampicillin, streptomycin, tetracycline and trimethoprim-sulfamethoxazole, 3 to ciprofloxacin and nalidixic acid, and 2 to ceftazidime and cefotaxime. In total, 15 (9.15%) *Salmonella* isolates were classified as multidrug-resistant (MDR); these belonged to *S.* Infantis (*n* = 3), *S.* Rissen (*n* = 3), *S.* Typhimurium M.V. (*n* = 3), *S.* Brandenburg (*n* = 2), *S.* Typhimurium (*n* = 2), *S.* Manhattan (*n* = 1) and *S.* Virchow (*n* = 1) (Table 5). MDR strains were recovered from shellfish (*n* = 11), mammals (*n* = 3) and water (*n* = 1). Specifically, 7 of the 15 strains displayed simultaneous resistance to ampicillin, streptomycin, tetracycline and trimethoprim-sulfamethoxazole (ASSuT). Three of the seven *S*. Typhimurium monophasic variant strains showed an ASSuT profile (Table 5).

## 3. Discussion

In the present study, a total of 164 *Salmonella* isolates collected in southern Italy from wild animals and the environment were analyzed and several subspecies were detected. *Enterica* was the dominant subspecies and was isolated in all animals except frogs. *Enterica* was the only subspecies detected in shellfish, as already reported by [16], while in the other samples, five non-*enterica* subspecies were found. In contrast to the study in [6], the prevalence of *enterica* subspecies in mammals was higher than that of non-*enterica* subspecies. However, the results of the present study are in line with those reported by [17], in which all *Salmonella* strains isolated from wild boar killed in the Campania region belonged to the subspecies *enterica*. Moreover, the results of the present study also agree with those reported in other studies, in which the dominant subspecies isolated from other mammals (*Vulpus vulpes, Martes* spp. and *Meles meles*) was *enterica* [18,19].

The subspecies *enterica* is principally associated with warm-blooded animals, but can also be found in cold-blooded animals [20]. Indeed, in the present study, two strains isolated from reptiles belonged to this subspecies. However, cold-blooded animals are more associated with non-*enterica* subspecies [20], and isolates collected from frogs, snails and reptiles mainly belonged to subspecies other than *enterica*. Among these, the subspecies *diarizonae* was frequently detected; this subspecies is increasingly associated with infections in humans, particularly after direct contact with reptiles or after the consumption of mutton [21], though the prevalence of this infection in Europe is still low. Other non-*enterica* subspecies were also detected (*S. salamae*, *S. arizonae*, *S*. *hountenae* and *S*. *indica*); although *S. salamae*, *S*. *arizonae*, *S*. *hountenae* and *S. indica* have a poor ability to invade host cells, it has been reported that they can cause infection in immunosuppressed subjects [20].

Serotyping identified 64 serovars. *S*. Napoli, *S.* Typhimurium, *S.* Enteritidis, *S.* Rissen and *S.* Derby were the most frequently found. *S.* Napoli is frequently detected in southern Italy and has been associated with human outbreaks [22]. Moreover, *S.* Typhimurium, *S.* Enteritidis and *S.* Derby, along with monophasic *S.* Typhimurium, *S.* Infantis, *S.* Newport, *S.* Stanley, *S.* Kentucky and *S.* Virchow, which were also detected in the present study, are frequently reported in human cases in Europe [13]. By contrast, *S.* Rissen is frequently reported in human infections in the United States of America and Asia [23], but not in the European Union.

The various *Salmonella* isolates were tested against 11 antibiotics, in order to evaluate the occurrence of resistant and multidrug-resistant strains. A total of 104 bacterial strains (63.41%) proved resistant to at least one antibiotic. The highest levels of resistance were found against streptomycin, trimethoprim-sulfamethoxazole, tetracycline, ciprofloxacin and ampicillin. These drugs are used as first-line treatments for infections in humans and animals. High levels of resistance against streptomycin have also been reported in swine [24]. Streptomycin is categorized by the World Organization for Animal Health (OIE) as a “veterinary critically important antimicrobial agent” [25], and therefore its use should be limited; however, it remains important for therapy in animals when there are no alternative antimicrobials [26]. Moreover, high levels of resistance to trimethoprim-sulfamethoxazole, tetracycline and ampicillin, which are widely used in veterinary medicine as first-line treatments in animal infections, are commonly reported among domestic animals [14,24]. However, as expected, the proportions of resistance to these antibiotics observed among strains isolated from domestic animals are higher than those observed in wild animals in the present study [14]. Moreover, 13 strains showed simultaneous resistance to ampicillin and trimethoprim-sulfamethoxazole, which are used as second-line therapies in humans who fail to respond to first-line antibiotics (e.g., in the case of infection caused by resistant bacteria) [27].

As fluoroquinolones constitute the gold standard for the treatment of invasive salmonellosis in humans, the resistance to ciprofloxacin that we observed is of particular concern. The level of resistance to ciprofloxacin was even higher than the levels commonly reported in human isolates [14,28]. Moreover, *Salmonella* serovars also showed resistance to nalidixic acid. Fortunately, however, co-resistance to both fluoroquinolones proved to be rare (1.83%). Co-resistance to the third-generation cephalosporins (cefotaxime and ceftazidime) was also low; the importance of this result lies in the fact that these antibiotics are used to treat human infections when fluoroquinolones are not recommended (e.g., during childhood infection).

High levels of resistance to sulfonamides, tetracyclines and ampicillin are also frequently reported in human *Salmonella* isolates [14], while these, along with resistance to fluoroquinolones, are also frequently reported in foodstuffs of animal origin [29,30].

In terms of source, 29 *Salmonella* serovars (50.88%) recovered from mammals were resistant to at least one antibiotic. The highest levels of resistance were found against trimethoprim-sulfamethoxazole, streptomycin and ciprofloxacin. In comparison with the results reported by [6], we found higher levels of resistance to streptomycin (28.07% vs. 8.5%), cefotaxime (7.02% vs. 1.8%) and nalidixic acid (5.26% vs. 1.8%), but lower levels of resistance to gentamicin (3.51% vs. 5.5%) and ampicillin (1.75% vs. 3.7%). The results of the present study are also in contrast with those of [31], who found a lower level of resistance in wild boars. The discrepancy between the different studies could be attributed to different serovars hosted by different wild boar populations [17].

In shellfish, the highest levels of antimicrobial resistance were observed not only to trimethoprim-sulfamethoxazole, streptomycin and ciprofloxacin, but also to ampicillin and tetracycline. The presence of resistant *Salmonella* isolates in shellfish may indicate the spread of antibiotic-resistant strains from human or animal feces to aquatic ecosystems [32]. The low occurrence of resistant strains in water, as detected in the present study, does not contradict this hypothesis. Indeed, shellfish, being filter-feeding organisms, can concentrate unicellular algae, bacteria and other contaminants diluted in the environment; for this reason, they are often used for a more accurate analysis of water pollution [33].

Moreover, 11 strains isolated from shellfish, 3 strains isolated from mammals, and 1 isolated from water showed a profile of MDR. Furthermore, three *S*. Typhimurium monophasic variant strains from shellfish displayed the ASSuT profile, which is a recognized alert resistance profile for this serovar [1]. These results are of particular concern because shellfish and wild boars are commonly eaten by humans, to whom resistant *Salmonella* strains may therefore be transferred, causing infections that are hard to treat [9].

The evaluation of the occurrence of antimicrobial-resistant strains in wild birds is of great importance, as the long-distance migrations of some birds can spread resistant bacteria to different environments [9,15]. Around 70% of *Salmonella* strains isolated from birds were resistant to at least one antibiotic. These results are in contrast with those of [34], in which none of the isolates from wild birds exhibited phenotypic resistance.

In the present study, the occurrence of *Salmonella* strains displaying resistance, especially to streptomycin, was also found in isolates from amphibians, snails and reptiles. Some reptiles, besides being kept as pets, are also consumed by humans, and in recent years, the demand for their meat has increased in the EU. As reptiles carry a variety of pathogens, direct contact with these animals and the consumption of their meat may constitute a public health risk [35]. Thus, evaluation of the prevalence and antimicrobial resistance of the pathogens hosted by reptiles is essential in order to understand the magnitude of the risk associated with contact with reptiles or the consumption of their meat.

## 4. Material and Methods

### 4.1. Bacterial Strains

A total of 164 *Salmonella* strains isolated from 2014 to 2019 in the Campania and Calabria regions of southern Italy were collected and serotyped at the *Salmonella* Typing Centre of the Campania Region (Ce.Ti.Sa.; Department of Food Microbiology, Istituto Zooprofilattico Sperimentale del Mezzogiorno, Portici, NA, Italy). The strains originated from: mammals (*Sus scrofa, n* = 48; *Vulpes vulpes, n =* 8; *Martes martes, n* = 4; *Meles meles, n =* 2; unidentified species, *n* = 1), shellfish (*Mytilus* spp., *n* = 40; *Solen marginatus, n* = 12; *Tapes decussatus, n =* 5; *Donax* spp., *n* = 1), birds (*Anatidae, n* = 3; *Ardeidae, n* = 3; birds of prey, *n* = 3; pigeons, *n* = 1), reptiles (snakes, *n* = 2; lizard, *n* = 1; *Caretta caretta, n* = 1; *Testudo* spp. *n* = 1; unspecified species, *n* = 4), land snails (*n* = 3), frogs (*n* = 2) and environment (waters, *n* = 19). Isolates were collected from the intestine, spleen, liver and/or lymph nodes of (i) animals (*Sus scrofa*) shot by official hunters and (ii) animals recovered dead from the environment by veterinary practitioners, owners, or law enforcement and presented to the Istituto Zooprofilattico del Mezzogiorno (IZSM) for diagnostic investigation. Those collected from water were isolated during environmental monitoring. Strains were stored at −20 °C in Microbanks™ (Pro-Lab Diagnostics, Neston, UK) until the analysis.

The strains were cultured in Trypticase Soy Agar 5% (Oxoid, Basingstoke, UK) and incubated for 24 h at 36 °C. Biochemical identification by means of a Vitek device (bioMerieux, Craponne, France) and PCR for the detection of the *inv*A gene were carried out for confirmatory purposes [36,37].

### 4.2. Serotyping

Serotyping was performed in accordance with the Kauffman–White scheme [38] by means of agglutination with specific anti-sera for O (Statens Serum Institute–DK) and H antigens (Difco, Franklin Lakes, NJ, USA). *Salmonella enterica* subspecies Typhimurium and Blockley, provided by the National Reference Laboratory for Salmonellosis (IZS, Padua, Italy), were used as quality control strains. The serological identification of the *S*. Typhimurium monophasic variant was confirmed through molecular assays [39].

### 4.3. Antibiotic Susceptibility Testing

The antimicrobial susceptibility of the isolates was determined by means of the disk diffusion method, in accordance with the Clinical and Laboratory Standards Institute (CLSI) recommendations. The following antibiotics (Oxoid, Basingstoke, UK, and Becton Dickinson, Mississauga, ON, Canada) were used: nalidixic acid (NAL, 30 μg ), ampicillin (AMP, 10 μg), chloramphenicol (CLO, 30 μg), gentamicin (GEN, 10 μg), tetracycline (TET, 30 μg), trimethoprim-sulfamethoxazole (SUL, 25 μg), ciprofloxacin (CIP, 5 μg), colistin sulfate (CL, 10 μg), ceftazidime (CAZ, 10 μg), streptomycin (STR, 10 μg) and cefotaxime (CEF, 30 μg).

A quality-control strain (*Escherichia coli* ATCC 25922) was included in the test. The breakpoint for the interpretation of resistance or susceptibility to each antibiotic was that of the CLSI standards. In the evaluation of the results, strains displaying intermediate resistance were regarded as resistant, while those displaying resistance to at least three antibiotic classes were considered multidrug-resistant (MDR) [40].

### 4.4. Statistical Analysis

The significance of the differences in the resistance of the *Salmonella* strains recovered from the various sources was assessed by means of the chi-square test (χ^²^) through the EpiInfo 7 software package (Centers for Disease Control and Prevention, Atlanta, GA, USA).

## 5. Conclusions

In conclusion, ARB are widespread in wildlife and the environment in Italy. Indeed, we found resistant and MDR *Salmonella* strains among the serovars isolated from wild animals and the environment. Specifically, the highest levels of resistance were observed toward streptomycin, trimethoprim-sulfamethoxazole, tetracycline, ciprofloxacin and ampicillin, antibiotics that are commonly used as first-line treatments for infections in humans and animals. The occurrence of resistant strains in animals without a history of previous exposure reinforces the idea that resistant strains and/or antibiotic residues are spread to the environment from animal-rearing facilities. Shellfish exhibited the highest levels of resistant strains, indicating that anthropogenic activities in southern Italy probably have a higher impact on marine ecosystems than on terrestrial ones. Moreover, as most of the animals considered in the present study are usually eaten by humans, the presence of ARB in them is a matter of great concern. However, further research on the molecular profiles of these ARB is needed in order to define the association among domestic animals, wildlife and humans with regard to the occurrence of resistant bacteria and resistant genes.

## Figures and Tables

**Table 1:** Number and distribution of *Salmonella* subspecies detected from amphibians (frogs), birds, the environment, mammals, shellfish, reptiles and snails.

Source	*Salmonella Enterica Subspecies*						Total
	*enterica*	*diarizonae*	*salamae*	*arizonae*	*houtenae*	*indica*	
Amphibians	-	2	-	-	-	-	2
Birds	9	1	-	-	-	-	10
Environment	16	2	1	-	-	-	19
Mammals	49	5	6	1	1	1	63
Shellfish	58	-	-	-	-	-	58
Snails	-	3	-	-	-	-	3
Reptiles	3	1	2	1	1	-	9
Total	135	15	9	2	2	1	164

**Table 2:** *Salmonella* serovars or antigenic profiles identified within the subspecies *enterica, diarizonae*, *salamae*, *houtenae*, *S*. *arizonae* and *indica* isolated from wild animals and the environment.

*S. enterica Subspecies*	Serotype or Antigenic Profile	N° of Isolates	N° of Resistant Isolates *
*enterica*	Napoli	13	2
*enterica*	Typhimurium	11	11
*enterica*	Enteritidis	9	3
*enterica*	Rissen	9	7
*enterica*	Derby	8	5
*enterica*	Typhimurium M.V.	7	7
*enterica*	Give	7	4
*enterica*	Fischerhuette	6	4
*enterica*	Kasenyi	6	1
*enterica*	Infantis	5	5
*enterica*	Brandenburg	4	3
*enterica*	Anatum	3	3
*enterica*	Livingstone	3	3
*enterica*	Muenster	3	2
*enterica*	Nottingham	3	1
*enterica*	Ball	2	0
*enterica*	Coeln	2	1
*enterica*	Panama	2	1
*enterica*	Goldcoast	2	1
*enterica*	London	2	1
*enterica*	Manhattan	2	1
*enterica*	Umbilo	2	0
*enterica*	Veneziana	2	0
*enterica*	Bredeney	1	1
*enterica*	Cerro	1	0
*enterica*	Eastbourne	1	0
*enterica*	Havana	1	0
*enterica*	Kentucky	1	0
*enterica*	Kottbus	1	1
*enterica*	Litchfield	1	1
*enterica*	Messina	1	0
*enterica*	Montevideo	1	0
*enterica*	Muenchen	1	1
*enterica*	Newport	1	1
*enterica*	Ohio	1	1
*enterica*	Pomona	1	1
*enterica*	Reading	1	0
*enterica*	Saintpaul	1	1
*enterica*	Stanley	1	0
*enterica*	Stanleyville	1	0
*enterica*	Tennessee	1	1
*enterica*	Thompson	1	1
*enterica*	Tinda	1	1
*enterica*	Virchow	1	1
*enterica*	Worthington	1	1
*diarizonae*	48:-:1,5	3	3
*diarizonae*	59:-:en,x,z15	2	2
*diarizonae*	65:-:z	2	2
*diarizonae*	O:35:r:z35	2	2
*diarizonae*	50:r:1,5	1	1
*diarizonae*	60:k:z	1	1
*diarizonae*	61:i:z53	1	1
*diarizonae*	61:k:1,5,7	1	1
*diarizonae*	65:z10:e,n,x,z15	1	0
*diarizonae*	P:38:eh:1,5	1	1
*salamae*	41:z:1,5	6	4
*salamae*	L:21:g,t:-	1	1
*salamae*	S:41:z:1,5	1	1
*salamae*	S II:13,22:z29:1,5	1	1
*houtenae*	38:z4z23:-	2	2
*arizonae*	48:z4,z23:-	1	0
*arizonae*	51:z4,z23:-	1	1
*indica*	Y:48:z10:1,5	1	1

* isolates resistant to one or more antibiotics; N°: numbers.

**Table 3:** Overall occurrence (n.) and percentage (%) of resistance to 11 antibiotics (ampicillin (Amp), cefotaxime (Cef), ceftazidime (Caz), nalidixic acid (Nal), ciprofloxacin (Cip), gentamicin (Gen), streptomycin (Str), chloramphenicol (Clo), colistin sulfate (Cl), trimethoprim-sulfamethoxazole (Sul) and tetracycline (Tet)) in *Salmonella* spp. from amphibians (frogs), birds, environment, mammals, shellfish, reptiles and snails.

Source	*β-lactams*			*Quinolones*		*Aminoglycosides*						
	Amp	Cef	Caz	Nal	Cip	Gen	Str	Clo	Cl	Sul	Tet	Suscept. ^1^
Amphibians	-	-	-	-	-	-	2 (100.0%)	-	-	-	-	-
Birds	1 (10.0%)	1 (10.0%)	1 (10.0%)	-	1 (10.0%)	1 (10.0%)	3 (30.0%)	-	-	2 (20.0%)	4 (40.0%)	3 (30.0%)
Environment	1 (5.3%)	1 (5.3%)	-	2 (10.5%)	1 (5.3%)	-	4 (21.0%)	-	-	-	2 (10.5%)	12 (63.1%)
Mammals	1 (1.6%)	4 (6.3%)	1 (1.6%)	3 (4.8%)	11 (17.5%)	2 (3.2%)	16 (25.4%)	2 (3.2%)	1 (1.6%)	17 (27.0%)	3 (4.8%)	28 (44.4%)
Reptiles	-	-	-	-	1 (11.1%)	-	7 (77.8%)	-	1 (11.1%)	2 (22.2%)	-	2 (22.2%)
Shellfish	15 (25.9%)	5 (8.6%)	1 (1.7%)	6 (10.3%)	10 (17.2%)	1 (1.7%)	21 (36.2%)	4 (6.9%)	-	16 (27.6%)	20 (34.5%)	15 (25.9%)
Snails	1 (33.3%)	-	-	1 (33.3%)	-	-	3 (100.0%)	-	-	1 (33.3%)	-	-
Total	19 (11.6%)	11 (6.7%)	3 (1.8%)	12 (7.3%)	24 (14.6%)	4 (2.4%)	56 (34.1%)	6 (3.7%)	2 (1.2%)	38 (23.2%)	29 (17.7%)	60 (36.6%)

^1^ Suscept. = strains sensitive to all antibiotics used.

**Table 4:** Occurrence (n.) of resistance profiles observed within the subspecies *enterica, diarizonae*, *salamae*, *houtenae*, *S*. *arizonae* and *indica* isolated from wildlife and the environment.

*Salmonella Subspecies*	R-Type	N° Isolates
*enterica;diarizonae; salamae; indica*	Str	29
*enterica; salamae*	Sul	13
*enterica*	Amp; Sul; Tet; Str	4
*enterica*	Cip	4
*enterica*	Amp; Sul	2
*enterica*	Amp; Tet; Str	2
*enterica; salamae*	Cef; Cip	2
*enterica; diarizonae; houtenae*	Sul; Str	2
*enterica*	Sul;Tet	2
*enterica*	Sul; Tet; Str	2
*enterica*	Amp; Cef;	2
*diarizonae*	Amp; Caz	2
*enterica*	Amp; Clo; Sul; Tet	2
*diarizonae*	Amp; Str	2
*enterica*	Amp; Cip; Clo; Sul; Tet	2
*enterica*	Amp; Cip; Sul; Tet; Str	2
*enterica*	Amp; Cip; Tet; Str	1
*enterica*	Cip; Sul;	1
*enterica*	Cip; Tet; Str	1
*enterica*	Cip;Tet	1
*enterica*	Cl	1
*arizonae*	Cl; Str	1
*enterica*	Gen; Tet	1
*enterica*	Tet	1
*enterica*	Cef; Cip; Clo; Caz	1
*enterica*	Cef; Cip; Str	1
*salamae*	Cef; Cip; Sul	1
*enterica*	Cef; Cip; Tet; Str	1
*salamae*	Cip	1
*salamae*	Cip; Str	1
*houtenae*	Cip; Sul; Str	1
*salamae*	Cip;Clo; Sul	1
*enterica*	Clo;Gen	1
*enterica*	Clo;Tet	1
*enterica*	Nal	1
*enterica*	Nal; Amp; Cef;	1
*enterica*	Amp; Cef;	1
*enterica*	Nal; Amp; Cef; Tet; Str	1
*enterica*	Amp; Cef; Clo; Caz;	1
*enterica*	Nal; Amp; Cef; Sul; Tet; Str	1
*enterica*	Nal; Cip	1
*enterica*	Nal; Cip; Sul;Tet	1
*enterica*	Nal; Cip;Gen	1
*enterica*	Nal; Tet	1
*enterica*	Nal; Tet	1
*diarizonae*	Nal; Sul; Str	1
*enterica; arizonae; diarizonae;* *salamae; indica; houtenae*	susceptible	60

Note. Amp: ampicillin; Cef: cefotaxime; Caz: ceftazidime; Nal: nalidixic acid; Cip: ciprofloxacin; Gen: gentamicin; Str: streptomycin; Clo: chloramphenicol; Cl: colistin sulfate, Sul: trimethoprim-sulfamethoxazole; Tet: tetracycline. N°: numbers.

**Table 5:** Source of isolation of *Salmonella* serovars resistant to at least three antibiotic classes (multidrug-resistant; MDR).

Source	*S. enterica* Serovar	MDR Profile
Shellfish	Brandenburg	Cip-Amp-Str-Sul-Tet
Shellfish	Brandenburg	Amp-Str-Sul-Tet
Water	Infantis	Nal-Amp-Cef-Tet
Mammals	Infantis	Nal-Amp-Caz-Cef-Str-Gen
Mammals	Infantis	Amp-Str-Sul-Tet
Mammals	Manhattan	Cip-Cef-Str-Tet
Shellfish	Rissen	Cip-Amp-Clo-Sul-Tet
Shellfish	Rissen	Nal-Amp-Cef-Str-Sul-Tet
Shellfish	Rissen	Amp-Clo-Sul-Tet
Shellfish	Typhimurium	Cip-Amp-Str-Tet
Shellfish	Typhimurium	Cip-Caz-Cef-Clo
Shellfish	Typhimurium M.V.	Amp-Str-Sul-Tet
Shellfish	Typhimurium M.V.	Amp-Str-Sul-Tet
Shellfish	Typhimurium M.V.	Amp-Str-Sul-Tet
Shellfish	Virchow	Nal-Amp-Cef-Str-Tet

## Data Availability

Not applicable.
